# Heat Transfer Enhancement by Hybrid Nano Additives—Graphene Nanoplatelets/Cellulose Nanocrystal for the Automobile Cooling System (Radiator)

**DOI:** 10.3390/nano13050808

**Published:** 2023-02-22

**Authors:** Chong Tak Yaw, S. P. Koh, M. Sandhya, K. Kadirgama, Sieh Kiong Tiong, D. Ramasamy, K. Sudhakar, M. Samykano, F. Benedict, Chung Hong Tan

**Affiliations:** 1 Institute of Sustainable Energy, Universiti Tenaga Nasional (The Energy University), Jalan Ikram-Uniten, Kajang 43000, Malaysia; 2 College of Engineering, Universiti Malaysia Pahang, Gambang 26300, Malaysia; 3 Advance Nano Coolant-Lubricant (ANCL), College of Engineering, Universiti Malaysia Pahang, Pekan 26600, Malaysia; 4 Faculty of Mechanical and Automotive Engineering Technology, Universiti Malaysia Pahang, Gambang 26300, Malaysia; 5 Centre for Research in Advanced Fluid and Processes, Universiti Malaysia Pahang, Pekan 26600, Malaysia; 6 No. 9, Jalan Meranti Jaya 12, Meranti Jaya Industrial Park, Puchong 47120, Malaysia

**Keywords:** CNC, coefficients, correlation, energy, GNPs, heat transfer, hybrid nanofluid, radiator

## Abstract

A radiator is used to remove a portion of the heat generated by a vehicle engine. It is challenging to efficiently maintain the heat transfer in an automotive cooling system even though both internal and external systems need enough time to keep pace with catching up with evolving engine technology advancements. The effectiveness of a unique hybrid’s heat transfer nanofluid was investigated in this study. The hybrid nanofluid was mainly composed of graphene nanoplatelets (GnP), and cellulose nanocrystals (CNC) nanoparticles suspended in a 40:60 ratio of distilled water and ethylene glycol. A counterflow radiator equipped with a test rig setup was used to evaluate the hybrid nano fluid’s thermal performance. According to the findings, the proposed GNP/CNC hybrid nanofluid performs better in relation to improving the efficiency of heat transfer of a vehicle radiator. The suggested hybrid nanofluid enhanced convective heat transfer coefficient by 51.91%, overall heat transfer coefficient by 46.72%, and pressure drop by 34.06% with respect to distilled water base fluid. Additionally, the radiator could reach a better CHTC with 0.01% hybrid nanofluid in the optimized radiator tube by the size reduction assessment using computational fluid analysis. In addition to downsizing the radiator tube and increasing cooling capacity over typical coolants, the radiator takes up less space and helps to lower the weight of a vehicle engine. As a result, the suggested unique hybrid graphene nanoplatelets/cellulose nanocrystal-based nanofluids perform better in heat transfer enhancement in automobiles.

## 1. Introduction

Engine cooling is currently the largest technical difficulty confronting sectors in the manufacturing, electrical, and automotive industries. Many industrial coolant systems, car sectors, air-conditioning, power generation, and chemical processes employ conventional coolants like water, motor oils, mineral oil, ethylene glycol-processing instruments, and optoelectronic systems [1,2]. Enhancing the rate of heat transfer of ordinary is one of the base fluid’s primary issues for current science and technology [3]. To boost thermal performance and cooling systems with the best thermal performance, temperature reduction, accurate operating ability, and automotive cooling systems, such as electronic circuit cooling, heat exchanger cooling, high reliability. As a result, scientists and researchers were drawn to concentrate on comparing the heat-transfer characteristics of solid particles to those of common basic fluids [4,5].

**Figure 1:**
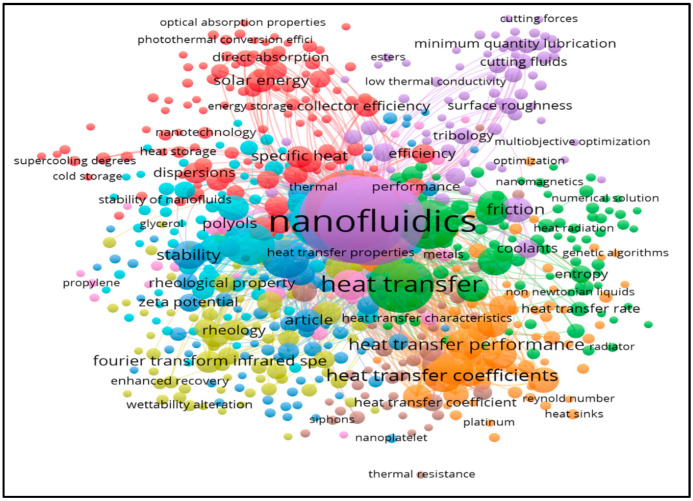
Magnetization Number of publications in graphene nanoparticles and bibliographic data of nanofluids from Scopus.

Maxwell [6] is the earliest to perform an experiment to increase the rate of heat transfer of micro-sized particles dispersed in common fluids, but the research was unsuccessful owing to sedimentation and flow pattern obstruction. Following multiple experiments, Choi et al. [7] discovered that suspending the presence of nano-sized particles in the host fluid can accelerate heat transmission; this fluid is known as nanofluid. Additionally, nanoparticle suspension enhances the base fluid’s stability while also improving the base fluid’s ability to transmit heat as compared to fluids containing milli- and micro-sized solid particles. Researchers are becoming increasingly interested in nanofluid applications since the creation of this new concept [8,9,10,11,12]. Now, to enhance modern automobile technology more efficiently and to be environmentally safe, new technological development is necessary [13]. Nevertheless, vehicles breaking down on the side of the road because they are too hot are typical nowadays. A deteriorating water pump and a worn-out radiator along with inefficient coolant are the main causes of engine overheating. Overheating can cause damage to different sections of the car, including cylinder head warping, radiator hose distortion, as well as a car breakdown [14]. There is nevertheless tremendous room for improvement, and numerous options are available. Improving car cooling system performance has been described. To improve cooling efficiency, there are three options: passive cooling, active cooling, and a mix of the two [15]. The engine’s running temperature should be kept under control to avoid mechanical problems in the materials caused by high operating temperatures, which allow for optimum fuel economy while preventing overcooling. In order to ensure the environmental condition, different writers have concentrated their research in recent years on increasing the radiator’s thermal performance by suggesting the utilization of cooling fluids with enhanced thermophysical properties. The effective method, on the other hand, mostly requires the use of external power to improve cooling abilities, like the installation of a cooling fan to eliminate extra heat or the use of an antifreeze (water and antifreeze) [16]. Equally, approaches are commonly employed by all vehicles for increasing and maximizing heat transfer efficiency. However, while maintaining the same system size, area, or input power, these solutions have limits. As a result, the addition of Nanoparticles for coolant, for example, has the potential to greatly enhance the effectiveness of the implicit cooling technique, because this innovation has been proven to significantly boost heat transfer performance in a wide range of subsystems [17,18]. The fluids in this category, known as nanofluids, are made after colloidal suspensions with solid nanoparticles (size 100 nanometers (nm)) in traditional coolant solutions. A total of several research articles were retrieved, along with their associated data, such as citations, author names, year, page, affiliation, keywords, title, and so on. Figure 1 shows the quantity of publications in graphene nanoparticles for research during the last 13 years, as well as the growth of the field. Figure 1 shows the bibliographic data of nanofluids from Scopus literature addressing the use of nanofluids in heat transfer application.

Several studies show that using nanofluids in automobile cooling systems can help to minimize the size and weight of the radiator while also collective efficiency and lowering fuel consumption [19]. To assess the performance of nanofluids, it is critical in vehicle cooling systems underneath an assortment of temperatures and situations to identify settings that increase the overall heat transfer coefficient along with reducing the amount of energy consumed in pumping [20]. In contrast, the bulk of applications where water is frequently utilized as the base fluid to create nanofluids in the literature involves combinations of water and ethylene glycol (at various ratios) and is also used in automotive cooling systems, given that the coolant fluid can reach temperatures of up to 200 °F [21,22]. To evaluate water-based alumina nano coolant, Gulhane et al. [23] used a fan-cooled radiator experimental setup. The factors that were modified were the nanoparticle concentration, flow rate, and temperature ranging from 0.1 to 0.4% (%), 2 to 5 L/min, and 50 to 70 °C. The heat transfer coefficient was increased by 45.87% with the baseline coolant’s 0.4 vol % Aluminium oxide nanoparticles. The authors recommended linear regression with a maximum variation of 3%.

Arunkumar et al. [24] investigated the performance of automobile radiators employing EG and water filled with various nanoparticles (Al_2_O_3_, MgO, and TiO_2_) as coolants. There were two unique concentrations of the nanoparticles (0.12% and 0.4%), at a ratio of 20:80(EG/W). The heat transfer coefficient was discovered to be linearly related to concentration, air, and nanofluid velocity. The behaviour of a copper-argon nanofluid contained in a nanochannel is investigated and the results imply that the surrounding structured liquid layers of the solid particles occupy a larger volume of the system in smaller channels, improving the thermal conductivity of the nanofluid [25,26,27,28]. Sharma et al. [29] investigated the impact of partial slip with graphene nanoparticles composed of EG. In the case of slip flow, it has been reported that increasing the fluid’s heating causes the Nusselt number to rise.

According to the author [14] instead of the Nusselt number calculation, the overall heat transfer coefficient (OHTC) to be provided in order of representing the heat transfer performance in a vehicle radiator This is since various Nusselt numbers are computed for all tubes or a single tube on the radiator. By adjusting the flow rate in the scope of 2–8 LPM, Naveen et al. [30] studied the heat transfer output of a radiator using graphene (70 W:30 EG) as a cooling medium and reported around 65% enhancement in the heat transfer rate and increment in the CHTC is 66% at a high flow rate. At increasing flow rates, the Nusselt number increases by 53.4%. Tijani et al. [31] investigated the thermal performance of an automobile radiator using Al_2_O_3_ and CuO nanoparticles at a 50-W:50-EG at 0.05, 0.15 and 0.3%. At 0.3% and 6 LPM, CuO-derived nanofluid illustrates a maximum heat transfer.

Hybrid nano coolants have also been proven to surpass mono-nano coolants in several investigations. Two or more types of nanoparticles are blended in with the base coolant to create a hybrid Nano coolant. Palaniappan et al. [32] did a study in which they combined a fly ash combination of many components (alumina, ferric oxide, titania, magnesia, silica and calcium oxide, sodium oxide, Sulphur trioxide, and potassium oxide) in a mixture of deionized water and ethylene glycol (DI/EG). A 6-cylinder IC engine and a crossflow radiator were part of their arrangement. The optimal concentration (2 vol %) of fly ash could increase the OHTC of base coolant, according to both energy and exergy calculations. Zadkhast [33] used MWCNT-CuO/water hybrid nanofluids to develop a new method for determining thermal conductivity. It was discovered that at 50 °C and a volume percentage of the thermal conductivity of nanofluid increased with an increase in nanoparticle content at 0.6%, with a maximum increase of around 30.38%. Jamshed et al. [34] examined features of hybrid nanofluids’ thermal transport comprising Cu and Zirconium dioxide nanoparticles derived from engine oil (EO). The numerical analysis found that compared to Cu-EO nanofluids, ZrO_2_-Cu/EO hybrid nanofluids have a higher rate of heat transmission. When compared to conventional nano coolants, the temperature of hybrid nanoparticle-based nanofluid rises faster [35].

Kumar et al. [36] examined two different hybrid kinds of nano coolants on an air-cooled radiator and identified that Al_2_O_3_-graphene nanoplatelet (GNP) Nano coolant outperformed Al_2_O_3_ carbon nanotube (CNT) Nano coolant by 2.94%. They concluded that the performance discrepancy was caused by the configuration of the carbon allotropes since GnP is a planar variety of carbon nanotubes with many layers. Further Sahoo [37] went on to improve the research by combining the three nanoparticles in water to generate an Al_2_O_3_-GnP-CNT trihybrid Nano coolant. The strong pumping power of the trihybrid Nano coolant is substantially higher than any of its ability to improve heat transfer, hence based on their research, it is not advised to be used in place of a coolant. Due to their superior thermal and heat transmission qualities, hybrid nanofluids have been the subject of several investigations in a variety of sectors. Table 1 lists some of the hybrid nanofluid applications. Due to the fact that hybrid nanofluids have remarkable heat transmission capabilities, they were used in this work to investigate their behavior in an automotive radiator. Table 1 gives a quick overview of how hybrid nanofluids are used in diverse applications. In addition to radiator exchangers, hybrid nanofluids and base fluids are anticipated to be important in a range of industries in the near future, including renewable energy, biomedical applications, sensor, compatibility, and so on [38,39,40,41].

The majority of the studies in the literature suggested that nanoparticles improve the heat transfer performance of base coolant or even reduce the size of a radiator, implying that they could be utilized as a replacement for traditional coolant. Various study scopes lead to several results on nano coolant’s better heat transfer performance. However, research into the application of hybrid Nano coolant in a car radiator is currently limited. Recent studies stated that hybrid nanoparticles can increase the thermal properties of a base fluid since conventional heat transfer fluids such as water and ethylene glycol exhibit very low thermal conductivity (aqueous ethylene glycol solution at 30 °C is 0.334 W/m-K). In this study, the author combines two types of nanoparticles, namely GnP with CNC in a volume fraction of 0.2%, and variations of nanoparticles ratio GnP:CNC is 50%:50%. While Ethylene glycol and distilled water are basic fluids to find out the variations in the physical characteristics of the heat transfer. The main objective of the current study is to improve the heat transfer performance of the radiator using novel hybrid nanofluids. This study used a radiator test rig to simulate a car cooling system to examine the thermal performance of graphene-based hybrid nano coolants with varying flow rates. The authors have previously published work on the characterization, preparation technique, stability, and thermophysical properties of the hybrid Nano coolant employed in this study [41].

## 2. Materials and Methods

### 2.1. Details of Nanofluids

**Figure 2:**
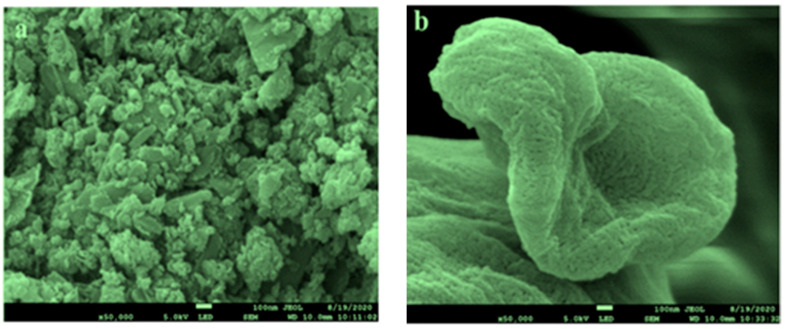
FESEM images of nanoparticles. (**a**) Graphene nanoplatelets; (**b**) Cellulose nanocrystal.

**Figure 3:**
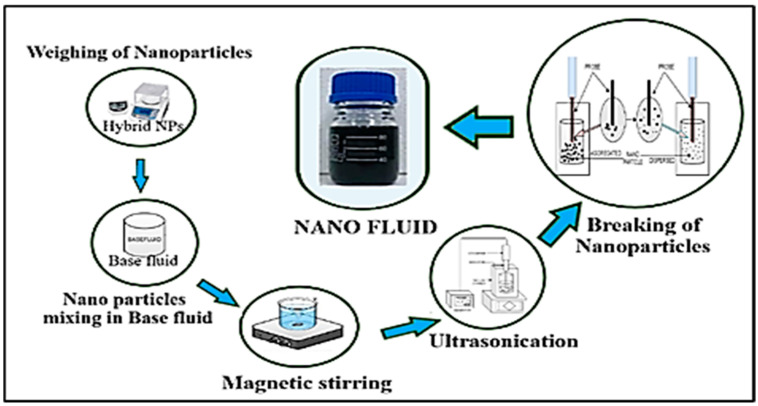
The nanofluid preparation.

Graphene nanoplatelets (GNP) with a purity of 99.9% (Nanografi Nanotech., Camsdorfer Ufer 12 07749 Jena, Germany) and used untreated CNC with 7.4% weight/weight suspension has been bought from Canada’s Blue Goose Bio-refineries Inc. As a base coolant, Distilled Water/Ethylene Glycol was blended in a 40:60 ratio. The base fluid contains nanoparticles, Graphene nanoplatelets (GNP), and cellulose nanocrystals (CNC). A total of 4 L of Nano coolant was created for the test by vigorous magnetic swirling for 120 min and ultrasonication for 5 h. FESEM images are shown in Figure 2. In order to prepare hybrid nanofluids, the two-step method has been applied and depicted in Figure 2. The physical properties and weighing are detailed and discussed in the previously published article [41]. Hybrid nanofluids preparation is depicted in Figure 3.

### 2.2. Details of Heat Transfer Equipment

**Figure 4:**
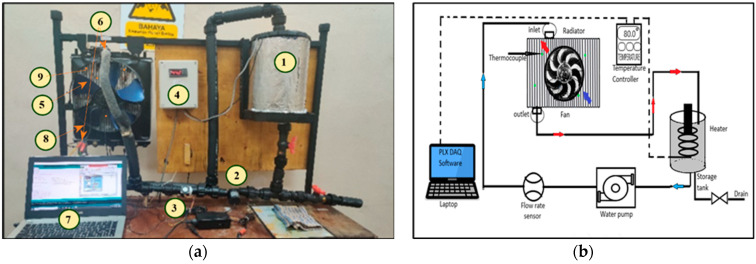
Heat transfer equipment. (**a**) The test bed; (**b**) The schematic.

Figure 4 shows a test bed and a schematic illustration of the research’s heat transfer test equipment. For the experimental run, the tank is full of 4 L of coolant. An electric heater to warm the fluid, LM-35 thermocouples, and K-type DS18B20 thermocouples are used to measure the surface and bulk temperatures of the radiator. An analog temperature sensor and flow rate sensors are used to monitor the temperature and flow. As the heat exchanger, a real automobile radiator (Perodua Kancil) with 31 tubes and 32 columns of serpentine fins was employed. The radiator has a major diameter, D is 0.022 mm, and a minor diameter, d of 0.002 mm with a length, L of 0.375 mm. Fins are made of up Aluminium with 1 mm thickness to enhance heat dissipation. The geometrical specifications of the radiator are defined and listed in Figure 4, Table 2 and Table 3, respectively. A brushless water pump, JT-800D is used to provide a constant flow rate of nanofluid to the radiator in a closed loop.

**Figure 5:**
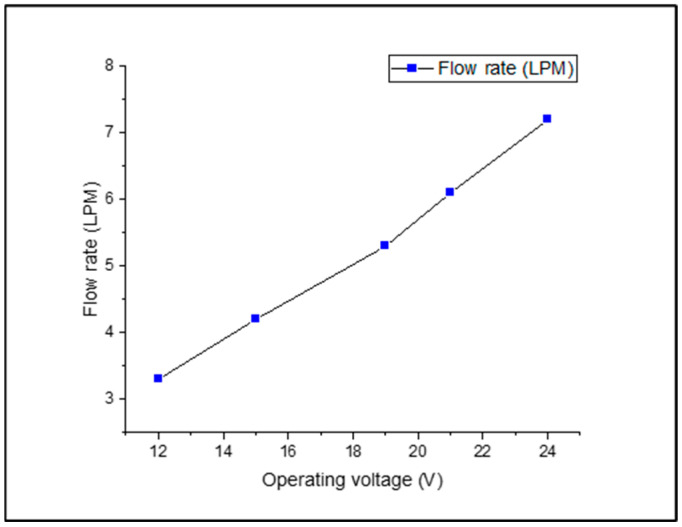
Measurement of flow rate at various operating voltages.

The coolant flow rate range was chosen based on previous experiments with crossflow type radiators. The coolant flow rate was set between 3.3 LPM to 7.2 LPM as in Figure 5 because the current study considered pump and Voltage limits. The temperature in the room is 27–30 °C and was utilized to keep the radiator cold in all the tests.

### 2.3. Experiment Procedure

The experiment starts with working fluid being fed into the radiator until it reaches a steady condition. The working fluid is then heated using a constant-power heater until the equilibrium temperature at the radiator inlet reaches 80 °C. This experiment is conducted under the influence of a radiator fan. A fan is employed to extract air from the radiator at a constant speed of 0.5 m/s. The experiment begins with a 3.3 LPM volumetric flow rate. The experiment is then repeated under identical conditions for volumetric flow rates of 4.3 LPM, 5.3 LPM, 6.3 LPM, and 7.3 LPM. With the use of PLX-DAQ connected with Microsoft Excel software, raw data acquisition for inlet temperature, outflow temperature, and surface temperature at four locations is obtained once a steady state is reached. Meanwhile, an infrared camera is utilized to photograph the radiator in order to analyze the temperature distribution profile on the radiator for each parameter and condition. for data verification purposes Four common laminar correlations and the well-known Dittus-Boelter equation were used to compare the Nusselt number [51,52,53,54], after obtaining a range of acceptable outcomes. Overall heat transfer coefficients of the radiator were compared. Finally, the information gathered is analyzed and discussed.

### 2.4. CFD Simulation Analysis and Governing Equations

**Figure 6:**
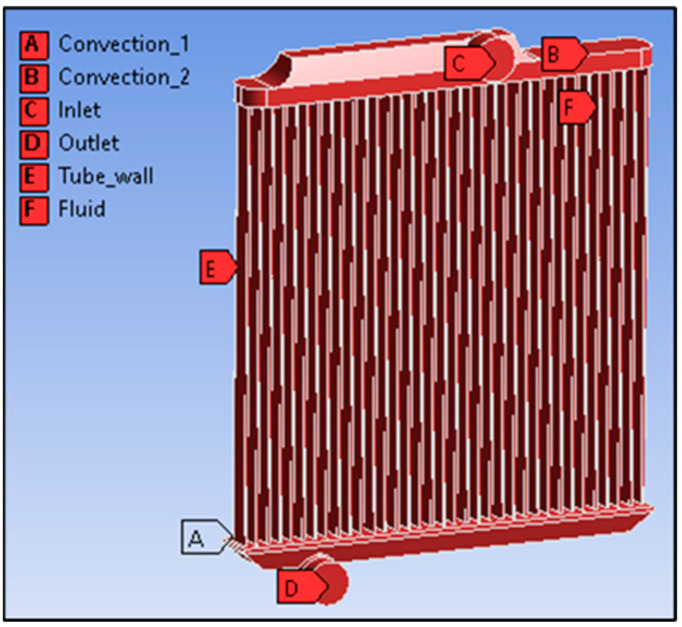
Measurement of the radiator model in simulation software (SOLIDWORKS 2020, USA).

The 3D model is designed using SOLIDWORKS 2020. The simulation process is performed in ANSYS FLUENT 2020 R1. The model is imported into the design modular in Geometry for extracting the 3D model into the simulation environment and the model is assigned as a fluid model. The mesh size of this model is 20 mm along with linear element order and solver preference as fluent. The mesh quality is assigned with target Skewness as 0.9 and smoothing is set to medium to increase the mesh quality and accuracy of the outcomes. The inflation is applied over the tubes to create mesh structures that are starting from faces or surfaces of geometric right inside to the below in layer format. The maximum layer of inflation is set to 10 and the growth rate is 1.2 per layer. Then, face meshing is assigned over the tube walls to map the face for smooth flow. The name selection is assigned for respected places such as inlet, outlet, convection 1, convection 2, Fluid domain, and tube wall. These name selections are assigned as per the below image Figure 6. In the mesh independent analysis, the mesh is obtained with 380,253 nodes and 298,520 elements for the high mesh and 462,015 nodes and 331,523 elements are obtained with the medium mesh setting. As such for accuracy and using tetrahedral meshes for curvature adaptations the high mesh is chosen.

Then, the ANSYS Fluent is run for the radiator simulation. The solver type is selected as pressure based with absolute velocity formulation and transient time. Gravity is assigned in the direction y-axis with −9.81 m/s^2^ for the downward direction. In models, energy is on for the simulation process and viscous type is selected as k-epsilon (2 Eqn) realizable model along with enhanced wall treatment. After that, the materials are created as per the proposed coolants in the material Table In cell zone condition, the respected fluid is assigned for the fluid domain. The stationary wall motion and no-slip requirement for the momentum at convection zones 1 and 2 are the major boundary conditions of the simulation used for the radiator analysis. Convection is listed as a thermal condition, and aluminum is the substance. The velocity magnitude (m/s) at the inlet named selection is described with input flow rates ranging from 3 lpm to 7 lpm, with a maximum inlet temperature of 348 K. In boundary condition, the inlet is set to velocity, the outlet is set to pressure, and convection 1 and 2 are set to convection thermal condition along with the respected coefficient of heat transfer value which is acquired because of the simulation procedure. The tube wall is also assigned to convection thermal conditions with a respected heat transfer coefficient value. In solution methods, the SIMPLE method is used with Green-Gauss node-based gradient and second order for pressure. Similarly, seconder order upwind is used in simulation methods such as momentum, turbulent kinetic energy, and turbulent dissipation rate. Thereafter, the setup is initialized, and the simulation is run for 100 iterations. In the results, the code is used for discovering the heat transfer value in the simulation area average (surface heat transfer coefficient)-tube wall.

CFD method practices numerical calculation by solving mass, momentum, and energy conservation governing equations as follows,

Continuity equation

(1) ∂ρ/∂t+∇_.(ρU)=0,

Momentum equation

(2) ∂/∂_t(ρU)+∇_.(ρUU)=−∇p+∇_.τ+ρg,

Energy equation

(3) ∂/∂t(ρh)+∇_.(ρUC_pT)=∇_.(k∇T),

### 2.5. Assumptions

Because of the reservoir tank’s heating and the radiator’s cooling, the temperature of the Nano coolant altered with the test rig. As a result of the thermophysical characteristics of Nano coolant, which dramatically changed due to differences in temperature, different performance measurements were altered. A few assumptions used by earlier researchers were used in this study to considerably simplify computations with minor errors.

- There is no or very little heat in the environment. Due to the insulated pipe external layer, the temperature gradient between the running coolant and the pipe wall surface is considered negligible. To attain a steady condition, the test rig is permitted to run for 30 min. The RTD PT-100 sensors are completely insulated from the inlet/outlet of the radiator. As a result, the bulk temperature (Tb) could be regarded as the average fluid temperature.
- In a continuous fluid flow, when operating circumstances remain constant, the flow rate and velocity of the coolant are expected to be constant over time. Furthermore, the excellent stability or homogeneity of the nano coolant indicates constant density and viscosity. The turbulence and temperature variations induced because of the adjacent flowing air are also reduced.
- Thermal dissipation among the outside and inside walls temperatures are projected to be negligible due to the modest radiator tube thickness (0.0002 m).

The following assumptions are made in resolving the analytical model:

There is no difference in changing the coolant flow rate. Effective thermal conductivity via the walls of the cooling tube is limited. Due to the fact that heat loss through coolant was solely delivered to the cooling air, no other heat transfer pathway, such as radiation, was addressed. Each tube’s coolant fluid flow has reached its full potential. The proportions are similar all through the radiator, and the heat transmission surface area is uniform and spread equally in all CFD Simulation and Heat Transfer Analysis of Automobile Radiator employing 31 Tubes. The radiator material’s heat conductivity is assumed to be constant. Within the radiator, there are no heat sources or sinks. Fluid stratification, losses, and flow misdistribution are not present. The tube wall is stationary at this time.

### 2.6. Data Reduction

Convection heat transfer is measured by Equations (4) and (5) as stated below to study heat transfer performance analysis [55].

(4) $$\dot{Q}C=nA\left(\Delta T\right)=nhA_{s}\left(T_{b}-T_{s}\right),$$

(5) $$\dot{Q}C=\dot{m}C_{p}(\Delta T)=\dot{m}C(T_{in}-T_{out}),$$

where *QC* = convection heat transfer (W), *h* = heat transfer coefficient (W/m^2^K), $T_{b}$ = bulk temperature (K), $T_{s}$ = surface temperature (K), *ṁ* = mass flow rate (kg/s), $C_{p}$ = specific heat capacity (J/kg.K).

The mass flow rate, *ṁ* (kg/s) is calculated by multiplying density, *ρ* (kg/m^3^), and volume flow rate, V̇ (m^3^/s) as shown in Equation (6).

(6) $$m^{\cdot }=\rho v^{\cdot },$$

Bulk temperature, $T_{b}$ is the mean of temperature inlet and temperature outlet as shown in Equation (7).

(7) $$T_{b}=(T_{in}+T_{out})/2,$$

where $T_{in}$ = temperature inlet (K) and $T_{out}$ = temperature outlet (K).

Surface temperature, $T_{s}$ is the wall temperature on the radiator surface and is calculated by averaging the temperature on it as shown in Equation (8).

(8) $$T\_s=1/4\sum ▒〖4/(i=1)T_{in}〗,$$

where $T_{in}$ = temperature inlet (K)

The experimental heat transfer coefficient, $h_{exp}$ is calculated by dividing Equation (9) by Equation (5) as shown in Equation (4).

(9) $$h_{exp}=\frac{\dot{m}C(T_{in}-T_{out})}{nAs(T_{b}-T_{s})},$$

Hydraulic diameter, Dh, is computed by using geometrical radiator dimension as shown in Equation (10). Major diameter, D, and minor diameter, d, are measured by using a digital calliper. The measured major diameter is 0.022 mm, and the minor diameter is 0.002 mm.

(10) $$D\_h=4(Area)/Perimeter=4[\pi /4d^{2}+(D-d)d]/(\pi d+2(D-d)),$$

Meanwhile, the Reynolds number and Nusselt number are calculated by using Equations (11) and (12).

(11) $$Re=(\rho vD_{h})/(nµ)=(4m^{\cdot })/(n\pi D_{h}µ),$$

where;

*Re* = Reynold’s number
*v* = flow velocity (m/s)
µ = dynamic viscosity (kg/ms)

The Nusselt number represents the ratio of convective heat transfer to convective heat transfer, which is another typical dimensionless measure used to represent heat transfer enhancement. Nusselt number is calculated as follows:

(12) $$RNu=〖h_{exp}D〗\_h/k,$$

where;

*Nu* = Nusselt number
*D_h_* = hydraulic diameter (m/s)
*k* = thermal conductivity (W/m. K)
*h_exp_* = Experimental heat transfer coefficient (W/m^2^K)

To verify the experimental data, the Nusselt number was estimated using Equation (12) related to the Nusselt number determined by means of association. The Nusselt number may be calculated using these formulae for both base and nano coolants. Meanwhile, the Nusselt number is defined by the Dittus-Boelter equation given below by Equation (13).

(13) Nu=0.023〖Re〗^(0.8)〖Pr〗^(0.4),

where *Re* and *Pr* are the Reynolds and Prandtl numbers calculated using the coolant’s bulk temperature Most researchers have examined their experimental results by using the Dittus-Boelter equation, which is suitable for internal turbulent flow in a single tube. For the verification of the data, some correlations that are dependable in much research for laminar flow are chosen. These correlations are expressed in the equations below [51,52,53,54].

Nusselt number correlation equation by [51].

(14) Nu=0.951〖Re〗^(0.173)〖Pr〗^(1/3),

Nusselt number correlation equation by [48] for compact heat exchangers.

(15) u=0.28〖Re〗^(0.35)〖Pr〗^(0.36)

Nusselt number correlation equation by [49] for the Reynolds number less than 33.33.

(16) Nu=1.953(Re.〖Pr⁡D_h/l_fin)〗^(1/3),

Meanwhile, the pressure drop is calculated as shown in Equation (17). Meanwhile, friction factor, f is calculated as shown in Equation (19) for turbulent flow using the Blasius equation, which is given below,

(17) $$△P=\rho gh=L/D.\rho u^{2}f.1/2,$$

where the above equation is derived from Equation (14),

△*P* = pressure drop (Pa)
*ρ* = density of fluid (kg/m^3^)
*g* = gravity acceleration (m/s^2^)
*h* = height of fluid column (m)

(18) $$f=(2△P)/(L/D\;\rho u^{2}),$$

(19) f=0.316/〖Re〗^0.25,

Equation (20) shows the calculation for heat transfer enhancement of thermal transport fluid in automotive radiators [56].

(20) E%=(〖Nu〗_nf−〖Nu〗_f)/〖Nu〗_f×100,

Finally, the overall performance of the automotive cooling system is influenced by thermal and hydraulic factors which are calculated as shown in Equation (21) [57].

(21) ƞ=((〖Nu〗_nf/〖Nu〗_f))/(f_nf/f_f)^(1⁄3),

The effectiveness of the radiator is calculated by using the equation below:

(22) $$Ɛ=(〖({\left(m\right)}^{\cdot }\_C\_(p))〗\_h(T_{in}-T_{out}))/(〖({\left(m\right)}^{\cdot }\_C\_(p))〗\_cT_{in}-T_{out}),$$

The overall heat transfer coefficient (U) based on air side surface area was calculated using the following Equation (23).

(23) $$U=1/(1/(n_{0}h_{a})+1/((A_{nf}/A_{a})h_{nf})),$$

### 2.7. Uncertainly Analysis

Using measured data such as temperature, mass flow rate, and thermophysical characteristics, the convective heat transfer coefficient, and overall heat transfer coefficient were calculated. The accuracy of the measurement instruments used determines the experiment’s uncertainty. Moffat et al. [58] noted that the accuracies of the measurement instruments were used to assess the experiment’s uncertainty. Uncertainty of the heat transfer rate (*δQ*) is calculated with the help of the derived equation below.

(24) $$\delta Q=\sqrt{{\left(\frac{\partial Q}{\partial \dot{m}}\delta \dot{m}\right)}^{2}+{\left(\frac{\partial Q}{\partial Cp}\delta Cp\right)}^{2}+{\left(\frac{\partial Q}{\partial T_{in}}\delta T_{in}\right)}^{2}+{\left(\frac{\partial Q}{\partial T_{out}}\delta T_{out}\right)}^{2}},$$

where,

*∂Q*/(*∂ṁ*) = *Cp* (*T_in_* − *T_out_*),
*∂Q*/*∂Cp* = *ṁ* (*T_in_* − *T_out_*),
*∂Q*/(*∂T_in_*) = *ṁ Cp*,
*∂Q*/(*∂T_out_*) = *ṁ Cp*,

Here, *δṁ*, *δCp*, *δT_in_*, and *δT_out_* are representing the uncertainties in mass flow rate, specific heat capacity, inlet temperature, and outlet temperature, respectively. The following derived equation is used to estimate the uncertainty in CHTC (*δh_exp_*).

(25) $$\delta h_{exp}=\sqrt{{\left(\frac{\partial h_{exp}}{\partial Q}\delta Q\right)}^{2}+{\left(\frac{\partial h_{exp}}{\partial A_{s}}\delta A_{s}\right)}^{2}+{\left(\frac{\partial h_{exp}}{\partial T_{b}}\delta T_{b}\right)}^{2}+{\left(\frac{\partial h_{exp}}{\partial T_{s}}\delta T_{s}\right)}^{2}},$$

where,

(*∂h_exp_*)/∂*Q* = 1/(A_s_ (*T_b_* − *T_s_*)),
(*∂h_exp_*)/(*∂A_s_*) = *Q*/((*T_b_* − *T_s_*)),
(*∂h_exp_*)/(*∂T_b_*) = *Q*/(*A_s_* (1 − *T_s_*)),
(*∂h_exp_*)/(*∂T_s_*) = *Q*/(*A_s_* (*T_b_* − 1))

Here, *δQ*, *δA_s_*, *δT_b_*, and *δT_s_* are denoting the uncertainty in heat transfer rate, surface area, bulk temperature, and surface temperature, respectively.

The uncertainty in the OHTC (U) was estimated using the following Equation (26).

(26) $$\delta U=\sqrt{{\left(\frac{\partial U}{\partial h_{a}}\delta h_{a}\right)}^{2}+{\left(\frac{\partial U}{\partial h_{nf}}\delta h_{nf}\right)}^{2}+{\left(\frac{\partial U}{\partial \eta_{O}}\delta \eta_{O}\right)}^{2}},$$

where,

(27) $$\delta \eta_{f}=\sqrt{{\left(\frac{\partial m}{\partial h_{o}}\right)}^{2}{\left(U_{m}\right)}^{2}},$$

The ambiguity in the gauging instruments and constraints is given in Table 4.

## 3. Results

**Figure 7:**
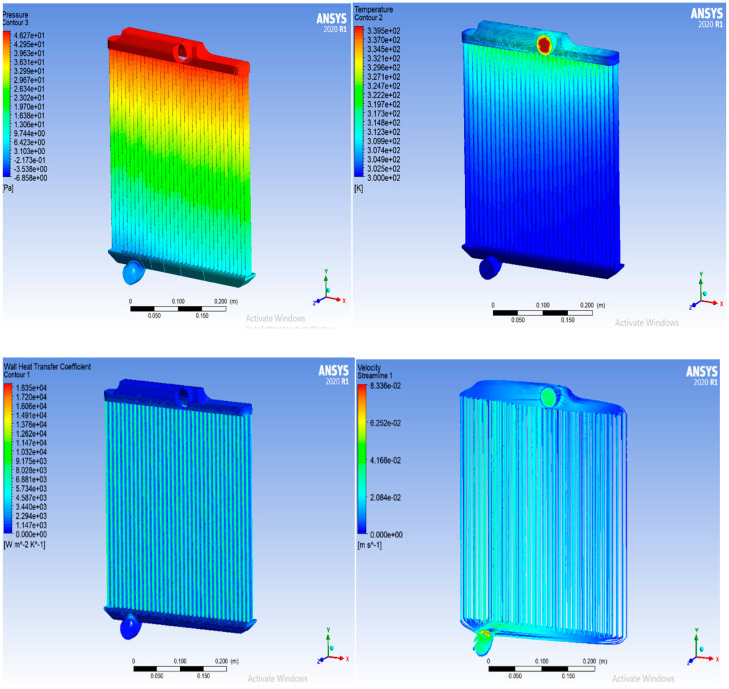
Radiator model temperature, pressure, velocity changes in simulation software.

Using various coolants such as distilled water (DW), ethylene glycol/water (60EG/40 W), and a proposed GNP/CNC hybrid nanofluid with 0.2% volume concentration. The pressure loss as a function of the heat exchanger is shown for a particle concentration of 0.2%. In every case, the greatest pressure gradient was detected at the heat exchanger’s input. Even though the pressure at the intake is equivalent to the pressure drop, the differential pressure between the inlet and outlet nozzle areas is visible. The temperature at the entrance point is extreme, as shown in the following photographs by the red color. The blue tint at the other end of the tube indicates that the hot fluid’s temperature has decreased while the hot fluid can pass through it. As shown in Figure 7, pressure and wall nearby temperature have similar observations of lowering temperatures. The steady-state velocity field for that iteration is represented by the velocity contour. Wall adjacent temperature is the temperature of the area closest to the wall, and it can only be plotted or presented at the wall. The most accessible temperature variable is the static temperature. The temperature at the wall and the temperature next to it are the same.

According to the data in Figure 7, as the coolant or working fluid flow rate in the radiator cooling system increases, the rate of heat transfer increases [59]. When the flow rate is larger, more heat energy is conducted from the coolant to the radiator flat tube. However, the coolant flow rate that can be used in an automobile cooling system has a limit. If the flow rate exceeds, it is important to prevent the flow rate restriction, aeration, or erosion on the radiator flat tube, and foaming of the coolant inside the system [60].

**Figure 8:**
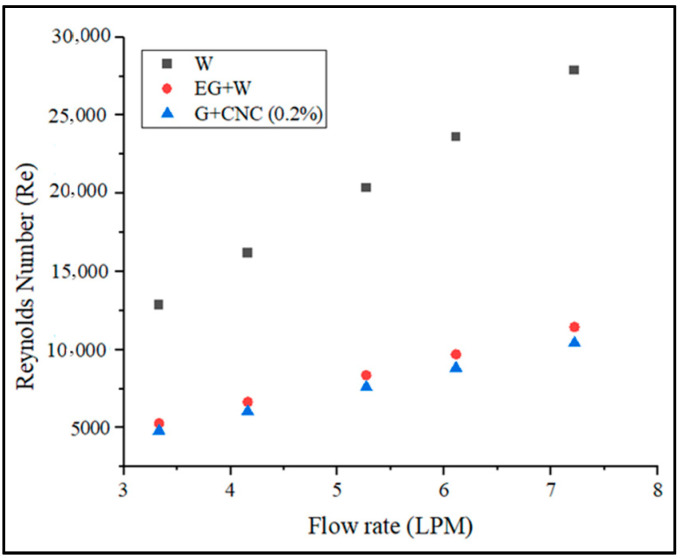
Reynolds number vs. flow rate in LPM.

In the current study, the Reynolds number is used to determine the nature of the flow pattern in the radiator test rig. Figure 8 displays a graph of Reynolds number vs. flow rate for water, base fluid, and 0.2% hybrid nanofluid.

**Figure 9:**
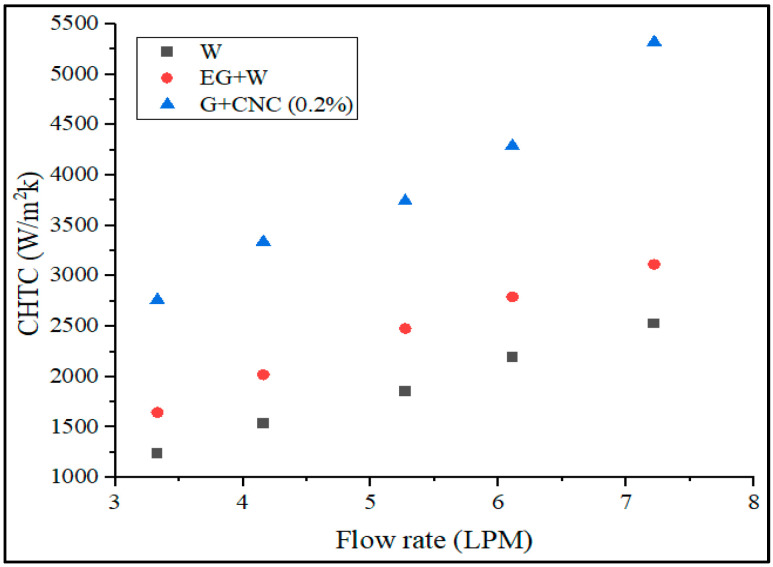
Convective heat transfer coefficient (CHTC) vs. flow rate in LPM.

According to the data collected, a directly proportional relationship exists between the Reynolds number value and the flow rate. The developed Reynolds number increases with the measured flow rate. Figure 8 demonstrates that as compared to water and base fluid, the suggested hybrid GNPs/CNC with 0.2% volume concentration has a lower Reynolds number. The figure also illustrates that when the flow rate increased, the Reynolds number value increased. Reynolds number was shown to drop with particle loading in the base fluid (0.2%) on account of increased density and viscosity, which results in a reduction in the mass flow rate. The result is, it is clear that particle loading causes a pressure drop to increase. As the flow rate increases, the Reynolds number also increases. The proposed GNPs/CNC has achieved 1786.37 and 3863.55 Reynolds numbers for flow rates 3.3 and 7.2 LPM, respectively. According to the data gathered, the flow rate and the Reynolds number value are directly proportional. The Reynolds number that develops increases with the measured flow rate. For a 3.3 LPM flow rate, the Reynolds number achieved is 4805.2 and at a 7.2 LPM flow rate, the proposed GNPs/CNC achieved Reynolds numbers of 10413.58, respectively. The resulting values can be used to demonstrate that the proposed fluid flow is turbulent. Convective heat transfer, as shown in Figure 9, is another important heat transfer performance analysis. The CHTC values obtained are plotted and illustrated for various flow rates.

**Figure 10:**
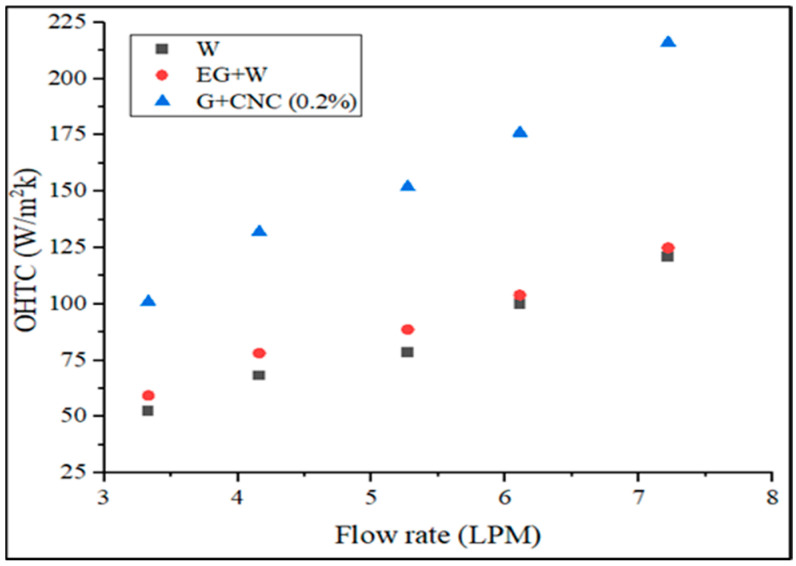
Overall heat transfer coefficient (OHTC) vs. flow rate in LPM.

Fluid properties, solid surface roughness, and fluid flow type together influence the convective heat transfer coefficient ‘h’ (laminar or turbulent). The proposed coolant performs well with maximum CHTC values at varied flow rates, as shown in Figure 9. The heat transfer coefficient still rises under the same conditions even when the Reynolds number is dropping fluid. This is a result of the loading of nanoparticles (0.2% GNP/CNC), improving thermal conductivity. Due to the fact that rising fluid temperatures induce a rapid rise in hybrid nanofluid’s heat transfer and an improvement in heat transfer coefficient are more noticeable at higher flow rates (6.43% at 3.3 LPM and 11.02% at 7.2 LPM with respect to base fluid) (due to enhancement in Brownian motion of nanoparticle). The proposed coolant achieved 2762.06 and 5318.22 W/m^2^K CHTC at 3.3 and 7.2 LPM flow rates, as related to base fluid EG/W, the suggested coolant CHTC value is 41.44% higher, and 52.46% higher when related to water at a flow rate of 7.2 LPM. Acquired overall heat transfer coefficient (OHTC) values in terms of various flow rates are shown in Figure 10.

**Figure 11:**
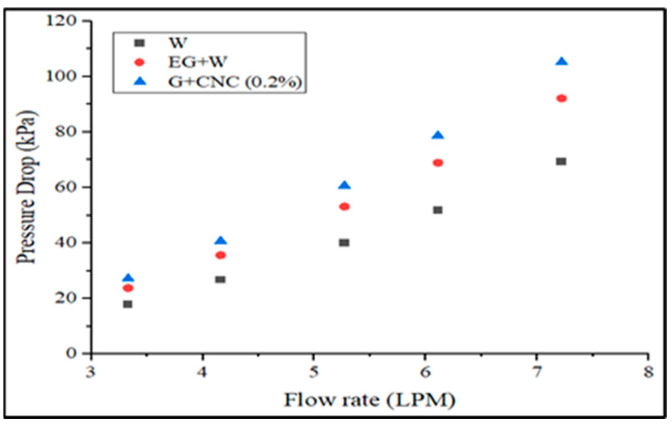
Pressure drop (kPa) vs. flow rate in LPM.

Figure 10 depicts the maximum OHTC values attained by the suggested coolant at various flow rates. Heat transfer coefficient still rises under the same conditions even when the Reynolds number is dropping (for hybrid fluid). This is a result of the loading of nanoparticles (0.2% GNP/CNC), improving thermal conductivity. Due to the rising fluid temperatures that induce a quick growth in the thermal conductivity of hybrid nanofluid, enhancement of heat transfer coefficient is more noticeable at higher flow rates (6.43% at 3.3 LPM and 11.02% at 7.2 LPM with respect to base fluid) (due to enhancement in Brownian motion of nanoparticle). Figure 11 displays the pressure drop of used coolants as a result of the flow rate.

**Figure 12:**
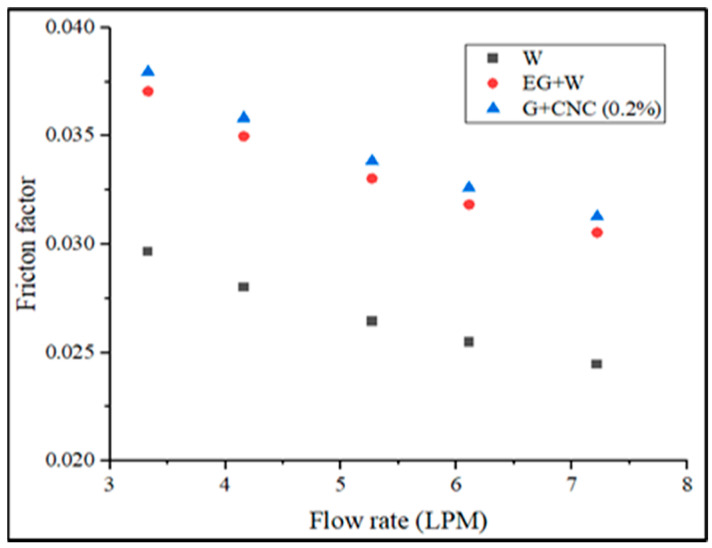
Friction factor vs. flow rate in LPM.

The proposed GNP/CNC obtained maximum pressure drop values with regard to various flow rates, as shown in Figure 11. Aside from the heat transfer coefficient, calculations of pressure drop are crucial in establishing the viability of applying hybrid nanofluids in operation. Reynolds number, density, and viscosity of the nanofluids govern the pressure drop inside the tube. The addition of hybrid nanoparticles to the base fluids increased pressure decrease. The pressure drop observed by the hybrid nanofluid at 0.2% concentration is 1.4 times higher than the pressure drop obtained by the base fluid (EG-W). This increase in pressure drop might well be explained by the fact that the base fluid’s viscosity rises as particles mix in the base fluid. The proposed GNP/CNC hybrid nanofluid pressure drop increased from 4.81 kPa to 18.55 kPa for 3.3 to 7.2 LPM flow rates, respectively. The pressure drop of the proposed GNP/CNC has increased by 12.35% when compared to base fluid (EG/W) and a 34.06% increase as compared with water at the flow rate of 7.2 LPM. The determined friction factor for executed coolants is shown in Figure 12 in terms of varied flow rates.

**Figure 13:**
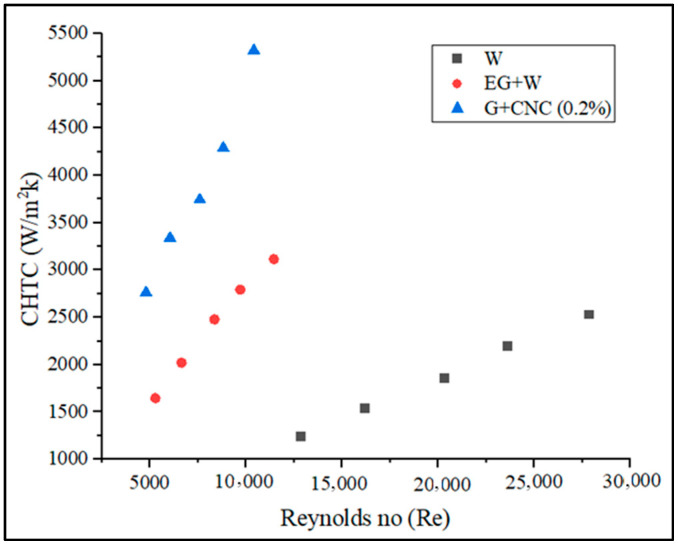
Convection heat transfer coefficient (CHTC) vs. Reynolds number (Re).

**Figure 14:**
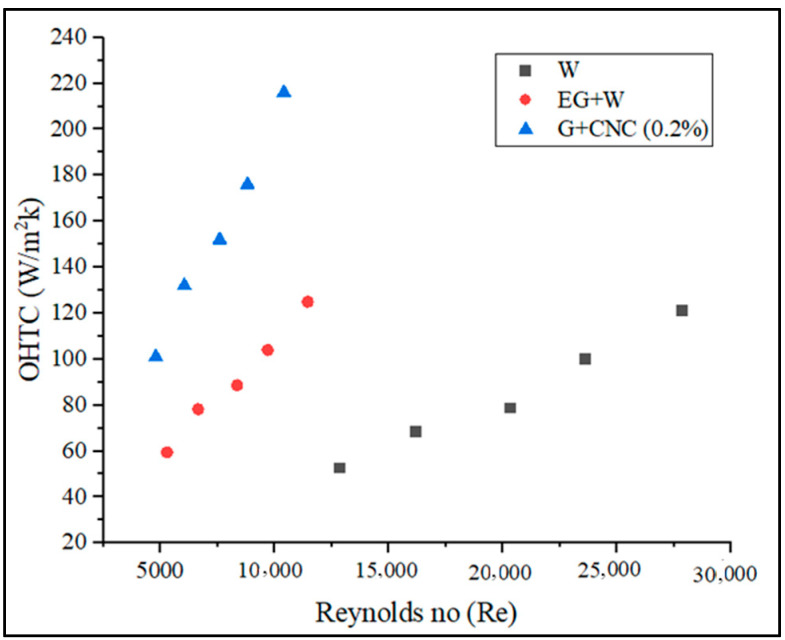
Overall heat transfer coefficient (OHTC) vs. Reynolds number (Re).

**Figure 15:**
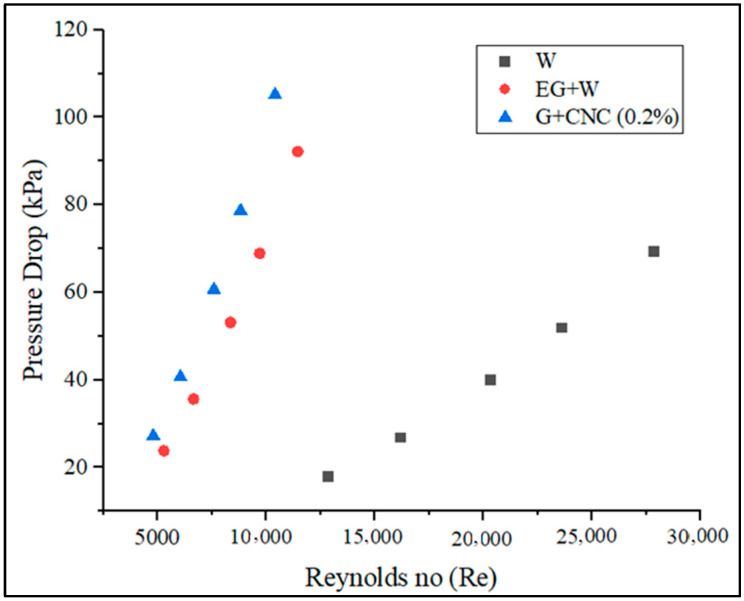
Pressure drop (kPa) vs. Reynolds number (Re).

**Figure 16:**
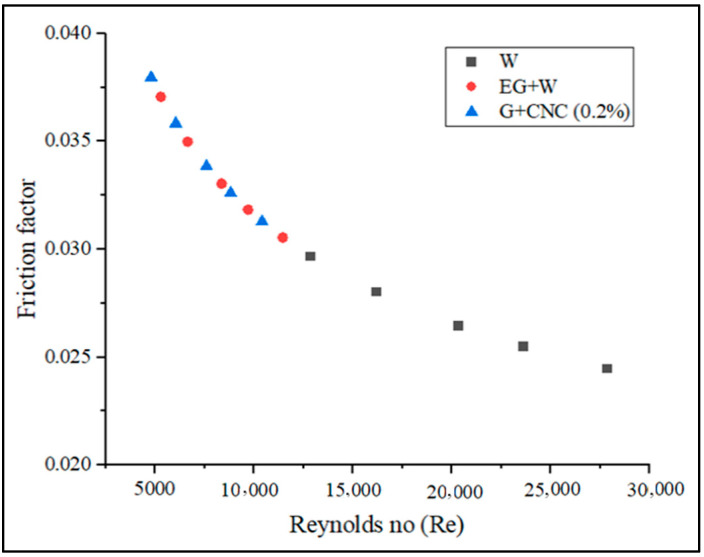
Friction factor vs. Reynolds number (Re).

**Figure 17:**
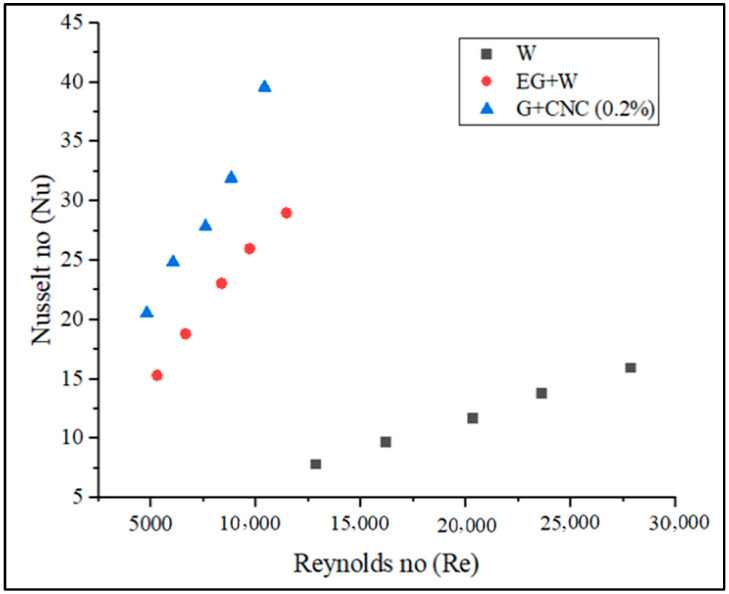
Nusselt number (Nu) vs. Reynolds number (Re).

The pressure drops seen between the outlet and the inlet of the radiator influences the friction factor. The suggested GNP/CNC has achieved maximum friction factor values with regard to various flow rates, as shown in Figure 12. As seen in Figure 12, the hybrid particle-based fluid raised the friction factor (f) in the radiator. The lowest friction factor value is measured for the water at the flow rate of 7 LPM. These findings are in line with those of research by Vajjha et al. [61]. The pressure drop between the flow’s inlet and outlet in a radiator determines the friction factor. The friction factor was plotted against the flow rate and Reynolds number for base fluids and hybrid fluids in this study. When there is relative motion between two bodies in contact, friction, a resistive force takes place. Three aspects primarily that determine the frictional force between two bodies are adhesion between the fluid and wall surfaces, the roughness of the surface, and the deformation factors. At a flow rate of 3.3 LPM for a hybrid nanofluid, the highest observed friction factor is 0.048, and the lower friction factor is observed at 0.040 at 7.2 LPM. Similarly, a 0.039 value is obtained for base fluid (EG-W). At the same flow rate, the lowest friction factor measured is 0.031 for water. The friction factor of the proposed GNP/CNC at 0.2% increased by 2.36% as compared with EG/W base fluid and by 21.82% increase when compared to Water base fluid at the flow rate of 7.2 LPM. The graphs drawn using CHTC, OHTC, pressure drop, friction factor, and Nusselt number in regard to Reynolds number are shown in Figure 13, Figure 14, Figure 15, Figure 16 and Figure 17.

Increased Reynolds number (Re) influences CHTC, OHTC, Pressure drops, and Nusselt number directly, according to Figure 13, Figure 14, Figure 15 and Figure 17. As the Reynolds number (Re) value improved, the value of the friction factor decreased. It is indeed apparent from the friction factor to the Reynolds number graph (Figure 16) that as the Reynolds number increased, the fluid’s friction factor also decreased dramatically. The water and base fluid achieved a lower friction factor in comparison to the hybrid GNP/CNC nanofluid. At a continuous air flow rate of 2 m/s, the Nusselt number varies according to the Reynolds number, and continual improvement is shown with 0.2% particle volume concentration and Reynolds number. This is because particle addition has increased the Prandtl number of the base fluid. Furthermore, because of the wide variability in the base fluids’ physical characteristics, it is impossible to directly connect the Nusselt number to the Reynolds number. Nusselt number for the proposed coolant has increased by 26.77% in comparison to base fluid (EG/W) and a 59.76% increase when related to water at the flow rate of 7.2 LPM.

**Figure 18:**
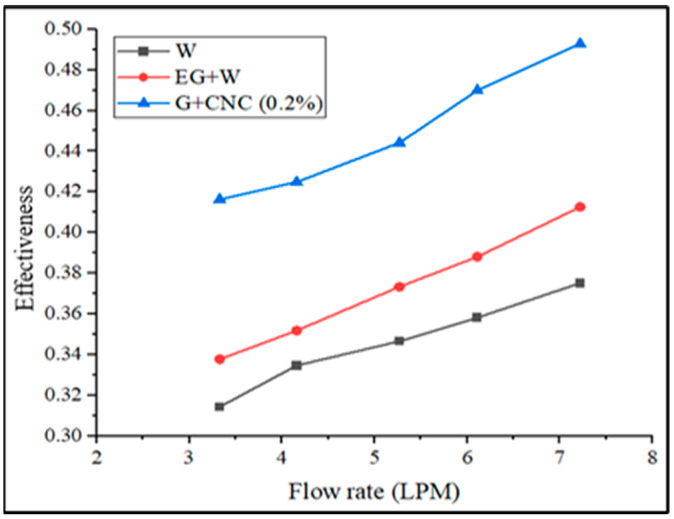
Effectiveness of automobile radiator.

**Figure 19:**
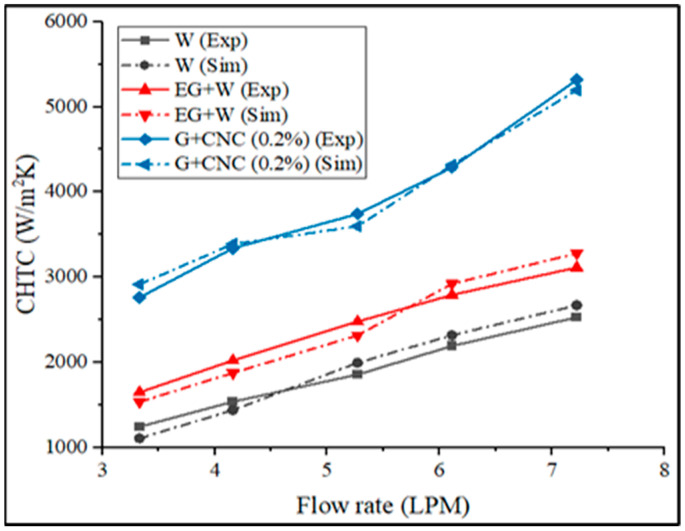
CHTC of experimental and simulation in terms of flow rate in LPM.

As the flow rate of the fluids increases, the radiator’s effectiveness at a fixed inlet flows temperature decreases. Similarly, as the flow rate increased, the nanoparticles mixed with the hybrid nanofluid increased. Figure 18 shows how the suggested GNP/CNC gains effectiveness at varied flow rates. The heat transfer improvement because of the addition of hybrid nanoparticles is shown in the pattern obtained from the effectiveness calculation. The effectiveness of the base fluid at 60:40 EG-W has obtained a 0.41 value at 7.2 lpm. The proposed GNP/CNC obtained a 0.49 effectiveness value for a 7.2 lpm flow rate. Overall, it was observed that hybrid nanoparticles’ heat transfer improves more than traditional coolants (water/EG-W) even at lower flow rate conditions. The results from the effectiveness calculations prove that the proposed GNP/CNC (0.2%) has achieved a better effectiveness value, and the maximum possible heat transfer can be possible with the hybrid nanoparticles with a 0.2% volume concentration. When it comes to the convective heat transfer coefficient (CHTC), Figure 19 illustrates the experimental and simulation results comparison.

The percentage of the difference between the experimental and simulation processes is assessed and marked in Figure 19. The difference between experimental and simulation results for the proposed GNP/CNC is 0.3%, 0.24%, and 0.3% for EG/W and water, respectively. The obtained calculated results demonstrate that the experimental and simulation results are consistent. The following sections correlate the experimental findings to the existing literature.

### 3.1. Comparative Analysis

According to the comparison analysis, the proposed GNP/CNC with 0.2% volume concentration performed well. It was also demonstrated that raising the flow rate and velocity of flow results in more efficient output. It can be determined that the proposed GNP/CNC hybrid nanofluid is more effective at removing heat from an automobile radiator. As an outcome of the combined effect of convective heat transfer and forced convection, and fluid influencing the rate of heat removal, increased efficiency is achieved. Heat transfer enhancement has a bigger impact than the frictional factor. Despite the fact that the friction factor in nanofluid is much higher than base fluid, the GNP/CNC hybrid nanofluid overall performance in the automotive radiator test rig is better than base fluid (EG/W).

**Figure 20:**
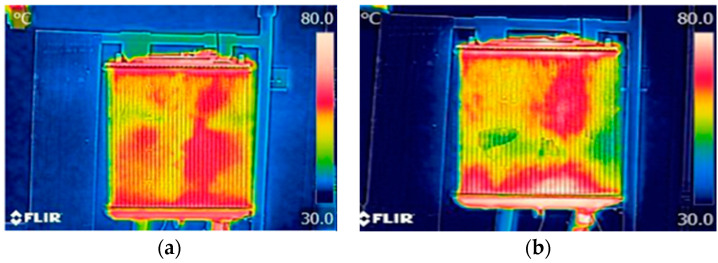
Thermal imaging of base fluids in the: (**a**) radiator; (**b**) nanofluids.

A thermal infrared camera was used to record the heat distribution of fluid inside the radiator. The temperature range is 30 °C to 80 °C, which is the radiator test rig’s optimal temperature. Thermal imaging of the fundamental fluid, which is distilled water and Ethylene Glycol, is shown on the left side of Figure 20.

Following the base fluids test, in the tank, a hybrid nanofluid with a volume concentration of 0.2% and a ratio of 60:40 (EG:W) was poured, and the experiment was then performed on the test rig to obtain a thermal image of the fluid exclusively in the radiator. The temperature range of nanofluid is the same as for base fluid, which is 30 °C to 80 °C. The colors yellow and green at the radiator are shown in Figure 20b. This observation in the image indicates that when the hybrid nanofluid runs in the test rig, it absorbs more heat. Hybrid nanofluids absorb more heat and enhance better than base fluids when compared to base fluids.

### 3.2. Size Reduction Analysis

This section used hybrid nanoparticles at different concentrations, GNP/CNC (0.2%), and other possible concentrations to get a more efficient heat transfer coefficient value. The actual and optimal radiator tube sizes are listed below in Table 5.

**Figure 21:**
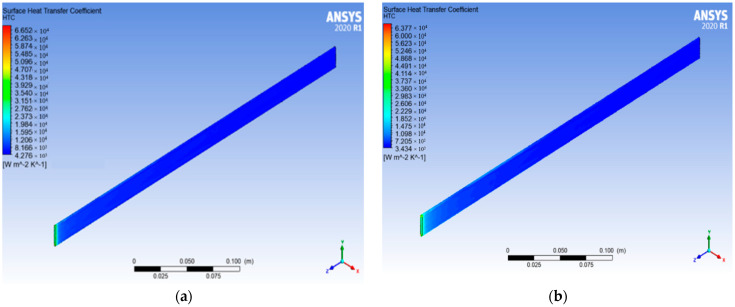
The pipe simulation at actual size: (**a**) G + CNC (0.2%); (**b**) G + CNC (0.01%).

The optimum size is determined by the CHTC value of GNP/CNC (0.2%) in actual tube diameter while keeping GNP/CNC constant (at 0.01% volume concentration). Figure 21 shows the surface HTC for actual tube sizes with GNP/CNC (0.2%) and GNP/CNC (0.01%).

**Figure 22:**
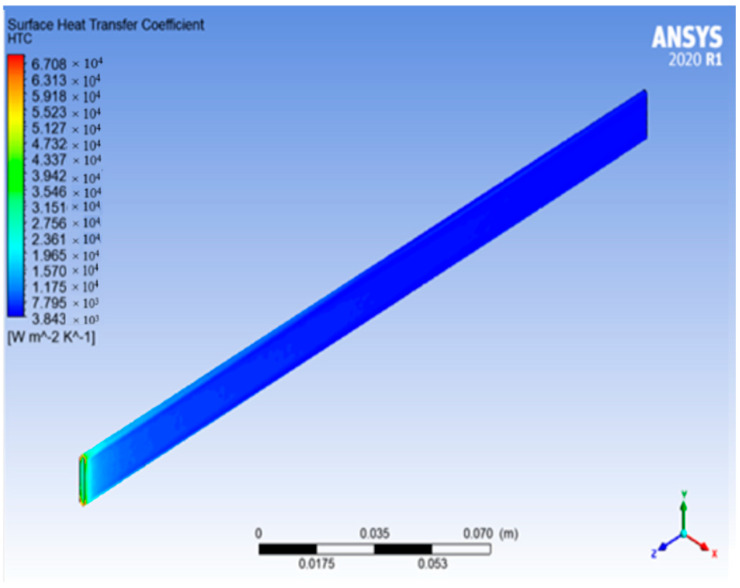
The pipe simulation at optimized size with G + CNC (0.01%).

According to the simulation images above, the actual radiator tube size achieves 6860.17 W/m^2^K for GNP/CNC (0.2%) and 5780.07 W/m^2^K for GNP/CNC (0.01%). The tube size analysis is performed first in order to reduce the radiator’s size, and the results for the surface HTC for GNP/CNC (0.01%) with optimized size are shown in Figure 22.

The surface HTC of an optimal tube size maintained with the hybrid nanofluid GNP/CNC at 0.01% volume concentration is 6661.21 W/m^2^K, which is close to the actual tube size with GNP/CNC at 0.2%, as seen in the above simulation image. With the optimized size of tube dimensions, the radiator may accomplish the Heat transfer coefficient value of actual tube size dimensions with a reduced volume concentration of hybrid nanofluid GNP/CNC at 0.01%, according to this simulation analysis by fluid flow. More space can be gained by shrinking radiator tubes, and greater heat transmission in the radiator can be attained with higher heat transfer coefficient values as nanoparticle concentrations in the base fluid are extremely low in volume.

### 3.3. GNP-CNC Hybrid Nanofluids Benefits for Heat Transfer

According to the mentioned experimental findings and analyses, the GNP-CNC hybrid nanofluids have a large thermal increase, which might significantly increase the efficiency of heat consumption. However, it was also necessarily followed by a rise in viscosity with volume concentration, which will have an influence on the time and energy required for pumping. The proposed hybrid nanofluid increased CHTC by 51.91%, OHTC by 46.72%, and pressure drop by 34.06% when compared to the existing hybrid nanofluid using distilled water as the base fluid. For distilled water and the suggested hybrid nanofluid, from the suggested dimensions, the CHTC value at 7.2 LPM is 2528.19 and 5318.22 W/m^2^K.

## 4. Discussion

This article looks into the effectiveness of an automotive radiator employing the Reynolds number, Nusselt number, CHTC, OHTC, pressure drop, and friction factor. The experiment is conducted at several flow rates, involving 3.3, 4.2, 5.3, 6.1, and 7.2 LPM. The outcomes of the experimental and simulation processes are discussed in this section. Furthermore, ANSYS Fluent 2020 R1 is used to carry out the simulation procedure. The numerical and graphical representations of computational simulation results can be studied (contour plot. In CFD post-processing, blue denotes the lowest value and red denotes the highest value [62].

**Figure 23:**
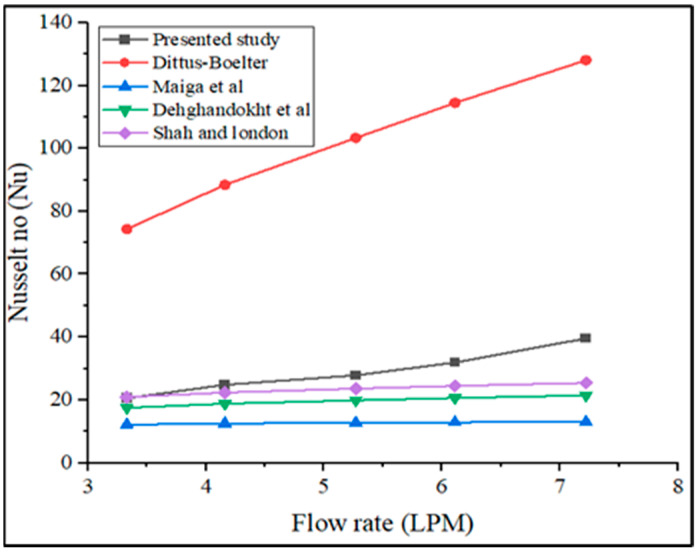
Comparison of Nusselt number of present studies with previous [51,52,53].

The obtained results from the experiments are validated by comparing them to previous literature. Figure 23 shows the comparison of obtained Nusselt number from experimental data with the Dittus-Boelter equation, which is widely used, along with Maiga et al. [51], Dehghandokht et al. [52], and Shah et al. [53] equations.

**Figure 24:**
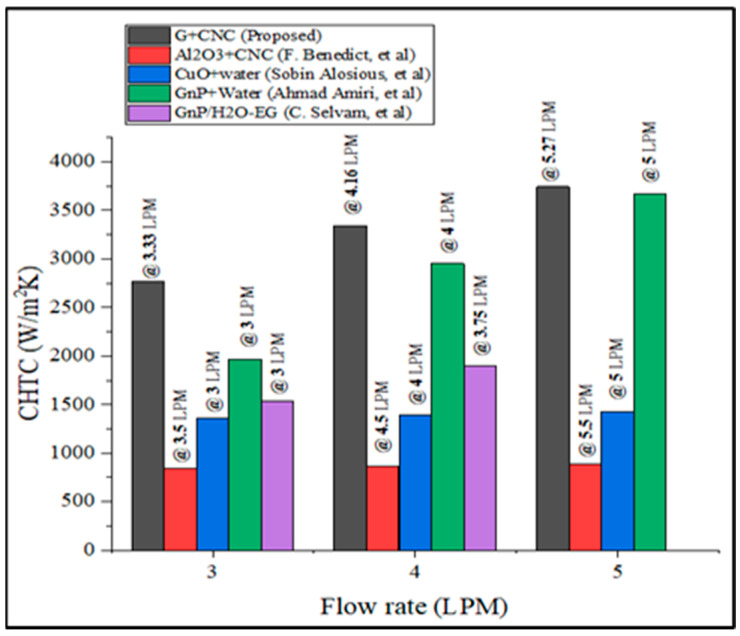
Comparison of CHTC of the present study with previous literature [63,64,65,66].

The Dittus-Boelter equation result obtained the highest Nusselt number in terms of various flow rates, as shown in Figure 23. When compared to Maiga et al. [51] equation, 49.86% when compared to the Dehghandokht et al. [52] equation, and 40.24% when compared to the Shah [53] equation with a flow rate of 7.2 LPM, the presented work experimental value is enhanced by 69.29%. Furthermore, the present research result has been reduced to 66.24% to the Dittus-Boelter equation. In addition, Figure 24 shows a comparison of the current study’s CHTC value with existing literature [63,64,65].

**Figure 25:**
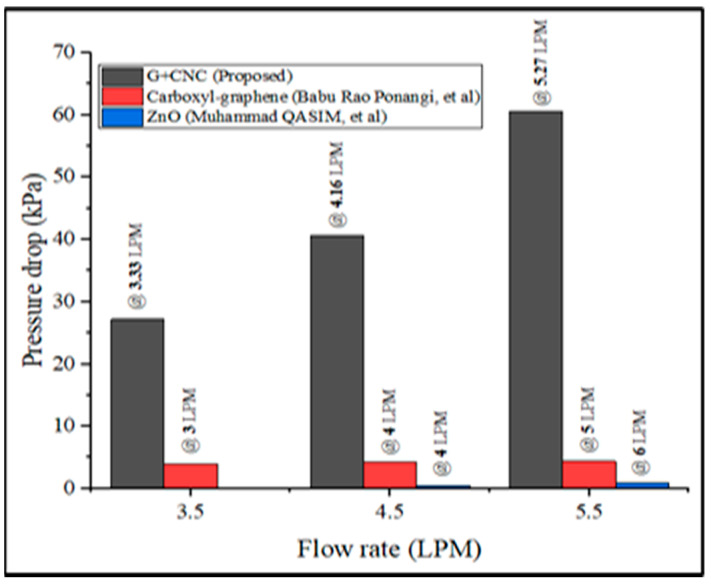
Comparison of pressure drop of the present study with previous literatures [67,68].

When compared to earlier literature, the preceding image reveals that the current study has attained the highest CHTC value. The comparison is made at three different flow rates: 3.5, 4.5, and 5.5 LPM. The CHTC value of the current study is increased by 76.38% when compared to Al_2_O_3_/CNC hybrid nanofluid, and by 60.82% when compared to Al_2_O_3_/water single nanofluid in the same research [63], with 61.79% improvement over the CuO/water single nanofluid [64], and 2.06% improvement over the GnP/Water single nanofluid research [64,65]. At a 4 LPM flow rate, the CHTC value of the proposed coolant is 43.19% higher than the GNP/H_2_O-EG nanofluid research [66]. Figure 25 shows a comparison of pressure drop with the literature [67,68].

In comparison to earlier literature, the current investigation shows a better performance in terms of pressure drop (Figure 25). For the comparison process, flow rates of 3.5, 4.5, and 5.5 LPM are used, and literature results are obtained from the author [68]. There is no data on thw pressure drop because this study is not conducted at 3.5 LPM. When compared to [67], an identical experimental setup, the pressure drop is 92.75% lower at 5.5 LPM, and 98.67% lower when compared to the author [68] data. The experimental technique was carried out in this study, with findings achieved at 70 °C and 0.2% zinc oxide nanoparticle concentration. The friction factor value obtained above 0.34 indicates the lowest level of pressure drop data obtained. The pressure drop value achieved by [67] at a volumetric concentration of 0.03% was low in comparison to the current proposed GNP/CNC (0.2%) investigation.

## 5. Conclusion

The primary goal and objective of this research is to improve the heat transfer coefficient of automobile radiators by using a novel hybrid nanofluid as a coolant. This work utilizes a mixture of graphene nanoplatelets and cellulose nanocrystals as a coolant for the radiator. The observations of the present study can be outlined as follows.

Heat transfer enhancement results using 0.2% novel hybrid nanofluid in car radiators are obtained. The experiment is carried out at various flow rates ranging from 3.3 to 7.2 LPM. From the previous studies of Stability analyses, thermophysical measurements, and RSM analysis results 0.2% hybrid nanofluid concentration acquires the best heat transfer property result. The results of the experimental test rig and a model created in 3D and evaluated by Computational fluid dynamics (CFD analysis in ANSYS software) conclude that enhancing the fluid’s flow rate increases the convective heat transfer coefficient (CHTC), overall heat transfer coefficient (OHTC), pressure drop, and Nusselt number. In comparison to the existing hybrid nanofluid, the suggested hybrid nanofluid enhanced CHTC by 51.91%, OHTC by 46.72%, and pressure drop by 34.06% with respect to distilled water base fluid comparison. The obtained Nusselt number agrees well with other correlations.

Furthermore, the recommended dimensions of a single radiator tube are 0.022 m major diameter, 0.002 m minor diameter, and 0.375 m length. At 7.2 LPM flow rate, these dimensions obtained 2528.19 and 5318.22 W/m^2^K CHTC for distilled water and proposed hybrid nanofluid, respectively. When the tube size “D” is reduced to 0.016 m and “L” is reduced to 0.185 m. The radiator could reach the CHTC of actual pipe size at hybrid 0.2% with 0.01% hybrid nanofluid in the optimized radiator tube, according to the size reduction assessment using CFD analysis. Furthermore, this demonstrates that by downsizing the radiator tube and increasing cooling capacity over typical coolants, the radiator takes up less space and helps to lower the weight of a vehicle engine. As a result, the suggested unique hybrid graphene nanoplatelets/cellulose nanocrystal-based nanofluids perform better in heat transfer enhancement in automobiles.

## 6. Future Perspectives

Few other applications with flow rates higher than 10 LPM can be examined further, even if there is a flow rate restriction in this study for a reason due to the nanoparticles being added to the base fluid, the current work can be expanded to analyze the wear and surface roughness aspects of radiator material. Graphene interactions with metal or metal oxide nanoparticles in nanocomposite powders have made it possible to design and create new applications including everything from energy problems to the medical industry. Therefore, it is essential to conduct additional research and develop newer processes for the preparation of graphene-based nanocomposites. The majority of experimental research has been carried out at room temperature but considering the wide range of applications for graphene-based nanofluids, it is equally crucial to assess their thermal properties characteristics at temperatures both higher and lower ambient levels. Future research should pay more attention to hybrid techniques that integrate continuous fluid dynamics and computation for researching nanofluids including hybrids. Examining the potential of refrigerants incorporating graphene to improve the thermal characteristics of condensers and evaporators used in two-phase heat transfer applications.

Radiator weight and size can be decreased by using the graphene family of nanofluids for cooling. They can also be used physically in the change to achieve heat transfer enhancement with the running load conditions. Finally, by addressing other enhanced representation investigations with the real experimentation application, the size reduction study employing CFD analysis in this research may be further expanded.

## Figures and Tables

**Table 1:** Several hybrid nanofluid applications and important findings.

Hybrid Nanoparticles	Volume Concentration	Application Area	Main Conclusion	Reference
Graphene nanoparticles/Al_2_O_3_/W	0.3%	Cooling of electronic devices	Nusselt number increases by 15% when compared to water.	[42]
Al_2_O_3_/Co_3_O_4_/W	0.015%/0.025%	Solar energy	80% nearer absorbance in radiation	[43]
Fe_3_O_4_/CNTs/W	0.1–1.35%	Double pipe heat mini channel	54% of heat transfer enhancement rate	[44]
Al_2_O_3_/MWCNTs/W	0.01%	Heat sinks mini channel	Increase in Convective Heat Transfer Coefficient by 44%.	[45]
MWCNTs/CopperOxide/W	0.03 wt%	Solar energy	Full absorbance of solar energy	[46]
Al_2_O_3_/Copper/W	1%	Heat sink micro fins	Nusselt number enhancement of 26% when compared to water. fins with a Diamond shape were crucial.	[47]
MWCNT/Al_2_O_3_/W	0.01%	Heat exchanger-plate	Increase in convective heat transfer coefficient by 15%	[48]
Silicon Carbide/MWCNT/EG	0.001–0.1 wt%	Solar collector	97% increase in thermal efficiency	[49]
Aluminum/Nitride/W	1.0–4.0%	Heat exchanger-double pipe 35%	35% of enhancement in thermal performance	[50]

**Table 2:** Components’ label from Figure 4a.

Label in Diagram	Part Name	Function
1	Electric heater	To increase the heat of the fluid.
2	Pump	To vary the flow rate for the radiator.
3	Flow rate Sensor	To measure flow rate.
4	Temperature Controller	To control temperature inlet.
5	Draft fan	Providing airflow to remove temp.
6	K-type DS18B20 Thermocouple	Used for measuring inlet/outlet of the radiator (bulk temperature).
7	PLX-DAQ	Software to record the temperatures.
8	Radiator	To remove heat from the operating fluid.
9	LM-35 Thermocouple	To determine the surface temperature of a radiator.

**Table 3:** Geometrical measurement for radiator and tube.

Details	Symbol	Configuration
Radiator height	h_rad_	450 mm
Radiator length	l_rad_	435 mm
Tube length	l_tube_	375 mm
Tube thickness	t_tube_	0.2 mm
Tube hydraulic (diameter)	D_h,tube_	3.73 mm
Number of tubes	N_tube_	31 no’s
The cross-sectional area of each tube	A_tube,cross_	0.0091304 m^2^
The outer surface area of each tube	A_tube,outer_	0.0174 m^2^
Tube and fin material	-	Aluminium
Fin height	h_fin_	0.0016 m
Fin length	l_fin_	0.0165 m

**Table 4:** Uncertainly in the measurement instruments and parameters.

Measuring Instruments/Parameters	Accuracy/Uncertainty
Temperature controller (STC-1000 Thermostat)	±0.1 °C
K-type waterproof thermocouple (DS18B20)	±0.5 °C
LM-35 thermocouple	±0.1 °C
Brushless water pump (JT-800D)	±0.1%
Uncertainty in heat transfer rate $\left(\delta Q\right)$	±3–7%
Uncertainty in CHTC $\left({\delta h}_{exp}\right)$	±5–10%
Uncertainty in CHTC $\left(\delta U\right)$	±6–10%

**Table 5:** Experimental dimensions of actual and optimized tub size.

Parameters	Actual (m)	Optimized (m)
D	0.022	0.016
d	0.002	0.002
L	0.375	0.24

## Data Availability

Not applicable.
